# SEPT12/SPAG4/LAMINB1 Complexes Are Required for Maintaining the Integrity of the Nuclear Envelope in Postmeiotic Male Germ Cells

**DOI:** 10.1371/journal.pone.0120722

**Published:** 2015-03-16

**Authors:** Chung-Hsin Yeh, Pao-Lin Kuo, Ya-Yun Wang, Ying-Yu Wu, Mei-Feng Chen, Ding-Yen Lin, Tsung-Hsuan Lai, Han-Sun Chiang, Ying-Hung Lin

**Affiliations:** 1 Division of Urology, Department of Surgery, Shin-Kong Wu Ho-Su Memorial Hospital, Taipei, Taiwan; 2 School of Medicine, Fu Jen Catholic University, New Taipei City, Taiwan; 3 Department of Obstetrics & Gynecology, National Cheng Kung University, College of Medicine, Tainan, Taiwan; 4 Graduate Institute of Basic Medicine, Fu Jen Catholic University, College of Medicine, New Taipei City, Taiwan; 5 Department of Medical Biotechnology and Laboratory Science, Chang Gung University, Tao-Yuan, Taiwan; 6 Institute of Bioinformatics and Biosignal Transduction, College of Bioscience and Biotechnology, National Cheng Kung University, Tainan, Taiwan; 7 Department of Obstetrics and Gynecology, Cathay General Hospital, Taipei City, Taiwan; 8 Institute of Systems Biology and Bioinformatics, National Central University, Zhongli City, Taoyuan County, Taiwan; Clermont-Ferrand Univ., FRANCE

## Abstract

Male infertility affects approximately 50% of all infertile couples. The male-related causes of intracytoplasmic sperm injection failure include the absence of sperm, immotile or immature sperm, and sperm with structural defects such as those caused by premature chromosomal condensation and DNA damage. Our previous studies based on a knockout mice model indicated that SEPT12 proteins are critical for the terminal morphological formation of sperm. *SEPT12* mutations in men result in teratozospermia and oligozospermia. In addition, the spermatozoa exhibit morphological defects of the head and tail, premature chromosomal condensation, and nuclear damage. However, the molecular functions of SEPT12 during spermatogenesis remain unclear. To determine the molecular functions of SEPT12, we applied a yeast 2-hybrid system to identify SEPT12 interactors. Seven proteins that interact with SEPT12 were identified: SEPT family proteins (SEPT4 and SEPT6), nuclear or nuclear membrane proteins (protamine 2, sperm-associated antigen 4, and NDC1 transmembrane nucleoproine), and sperm-related structural proteins (pericentriolar material 1 and obscurin-like 1). Sperm-associated antigen 4 (SPAG4; also known as SUN4) belongs to the SUN family of proteins and acts as a linker protein between nucleoskeleton and cytoskeleton proteins and localizes in the nuclear membrane. We determined that SEPT12 interacts with SPAG4 in a male germ cell line through coimmunoprecipitation. During human spermiogenesis, SEPT12 is colocalized with SPAG4 near the nuclear periphery in round spermatids and in the centrosome region in elongating spermatids. Furthermore, we observed that SEPT12/SPAG4/LAMINB1 formed complexes and were coexpressed in the nuclear periphery of round spermatids. In addition, mutated SEPT12, which was screened from an infertile man, affected the integration of these nuclear envelope complexes through coimmunoprecipitation. This was the first study that suggested that SEPT proteins link to the SUN/LAMIN complexes during the formation of nuclear envelopes and are involved in the development of postmeiotic germ cells.

## Introduction

Between 2% and 12% of couples worldwide are affected by infertility; in approximately half of these cases, the defects can be traced to the men [1]. The development of intracytoplasmic sperm injection (ICSI) in the past 2 decades has changed the treatment of extreme subfertility in men [2]. However, although ICSI constitutes a breakthrough in assisted reproduction, numerous infertile couples remain unable to achieve parenthood, even after using testicular sperm extraction. The causes of infertility are unknown in 25%–30% of men with spermatogenic failure, and deficient spermatogenesis is unresponsive to any conventional mode of therapy [3]. The sperm-related causes of ICSI failure include the absence of sperm, immotile or immature sperm, sperm with structural defects, premature chromosomal condensation, and DNA damage [4]. The loss of the nuclear integrity of sperm is not only associated with ICSI failure or poor fertilization following in vitro fertilization (IVF) but also linked to the developmental arrest of preimplantation embryos and high rates of miscarriage [2,4].

### SEPTs

SEPTs are highly conserved polymerizing GTP-binding proteins that belong to the fourth component of the cytoskeleton [5]. The functions of SEPTs are diverse, including establishing cell polarity, cytoskeletal remodeling, membrane compartmentalization, cell cycle progression, and vesicle trafficking, and are performed through interactions with several cytoskeletal proteins (e.g., actin, myosin II, and tubulins) [5]. SEPT proteins form homomeric and heteromeric filament-like structures in cells, such as the SEPT2/6/7 complex [6]. The loss of one component in a complex may cause the levels of other septins to decrease [7]. Some of the 14 members of the septin gene family in mammalian species are expressed ubiquitously, whereas others are expressed only in well-differentiated cells (e.g., neurons or male germ cells) [8]. Recent studies have demonstrated the crucial role of septins in various pathological processes including Alzheimer’s disease, hereditary neuralgic amyotrophy, leukemia, ovarian tumors, breast cancer, and male infertility [9,10]

#### SEPTs in spermatogenesis

SEPT4 is located at the annulus, a ring-like structure connecting the midpiece and the principal piece of the flagellum, and is vital for maintaining the proper mitochondrial architecture of the mid-piece and the integrity of the annulus in the sperm tail [11,12]. *Sept4* ^*-/-*^ mice were viable, but the males were sterile, with immotile sperm exhibiting a defective annulus. In humans, septins (SEPT1/4/6/7) are lost in most spermatozoa of asthenozoospermia patients [11,13,14]. We recently identified *SEPT12* as a potential sterile gene by employing a cDNA microarray analysis of the testicular tissue and found that SEPT12 was expressed in postmeiotic male germ cells [15,16]. Furthermore, mice with a deficient *Sept12* allele exhibited distinctive morphological defects (e.g., immature sperm head, bent tail, premature chromosomal condensation, and nuclear damage) [17]. In humans, *SEPTIN12* mutations and genetic variants in infertile men result in teratozospermia and oligozospermia [18–20].

#### SUN proteins involved in mammalian spermatogenesis

In all eukaryotic cells, the nuclear envelope (NE), which separates the nucleus from the cytoplasm, is composed of an outer nuclear membrane and an inner nuclear membrane [21]. SUN1 and SUN2, which belong to the SUN family, interact with LAMIN through N-terminal domains and connect to cytoplasmic actin by direct interaction with nesprin [22–24]. SUN1 and SUN2 knockout mice exhibited disruption of the link between the telomere and the NE, increased DNA double-strand breakage and apoptosis signals, decreased piRNA expression, and a resulting maturation arrest in the meiotic stage during spermatogenesis and oogenesis [25,26]. SUN3, SPAG4/SUN4, and SPAG4-like/SUN5 proteins have recently been included in this family [27–29]. SUN3 is a testis-specific protein that is expressed on the manchette, which is a transient structure present during spermiogenesis and involved in the shaping of the sperm head [27]. SPAG4 (SUN4) was identified using a yeast 2-hybrid system with outer dense-fiber protein 1 (Odf1) as bait and localized to the microtubules of the manchette and axoneme during spermiogenesis in rats [30]. In addition, SPAG4(SUN4)-GFP localized to the NE and endoplasmic reticulum (ER) in transiently transfected HeLa cells [31]. In *Drosophila*, knockout of SPAG4 disturbed the sperm head morphology and caused dissociation of the centrioles from the nucleus, resulting in male infertility [28]. SUN5/SPAG4L were expressed in meiosis stages I and II and localized in the nuclear membrane of HeLa cells [29].

To analyze the possible molecular functions of SEPT12 during spermiogenesis and the causes of spermatogenic defects in men with mutated SEPT12, we used a yeast 2-hybrid system to identify SEPT12 interactors. Among the interactor proteins was SPAG4 (SUN4). SEPT12/SPAG4/LAMINB1 formed complexes and were coexpressed in the nuclear periphery of round spermatids and in the sperm neck and centrosome regions of elongated spermatids during human spermiogensis. In addition, mutated SEPT12 screened from infertile men disrupted the integration of SEPT12/SPAG4/LAMIN complexes. On the basis of these results, we suggest that the optimal expression and interaction among SEPT12, SPAG4, and LAMINB1 is critical for sperm head formation during human spermiogenesis.

## Materials and Methods

### Yeast 2-hybrid screening and *β*-galactosidase assay

DNA fragments encoding the full-length human SEPT12 protein were generated from a human testis cDNA library (Clontech, USA) by using RT-PCR and subsequently inserted into a pBTM116 vector to produce baits for yeast 2-hybrid studies. This construct was used for screening in a human testis cDNA librry (Clontech, USA). Yeast 2-hybrid screening was performed as described previously [32]. The library plasmids conferred were then subjected to a DNA sequence analysis. Quantitative X-gal assays were performed using yeasts containing pairs of baits and prey plasmids. The X-gal activities were determined using liquid yeast cultures according to the instructions provided with the Galacto-light Plus kit (Tropix, USA).

### Reverse transcription-PCR (RT-PCR)

A human total RNA panel (Clontech, USA) was used to study the expression patterns of proteins that interact with SEPT12. The PCR conditions, DNA product detections, and control primer described in our previous publication were used [33].

### Preparation of human testicular spermatogenic cells and spermatozoa

This study was approved by the Institutional Review Board of Cathy General Hospital, and informed consent forms were signed by all enrollees. For an immunofluorescence assay (IFA), human normal testicular tissues (US Biomax, USA) and human ejaculated spermatozoa donated from fertile men were collected. Briefly, the testicular tissues were digested by enzymes and the suspensions were filtered through 35-μM nylon filters (Falcon; Becton Dickinson, Franklin Lakes, NJ, USA), and then centrifuged using a Kubota centrifuge 3330 (Kubota Corp., Tokyo, Japan) [34–35]. Germ cells at various developmental stages were collected. Semen samples were donated from 2 fertile men. Semen parameters were listed (Sample 1: sperm count, 20 × 10^6^; sperm motility, 65%; normal morphology, 31%; Sample 2: sperm count, 20 × 10^6^; sperm motility, 50%; normal morphology, 47%).

### Immunofluorescence assay

For an IFA, human germ cells were treated with 0.1% Triton X-100, washed twice with Tris-buffered saline (TBS), and subsequently incubated with a primary antibody (PRM2: Santa Cruz, sc-23104; NDC1: Santa Cruz, sc-161929; PCM1: Santa Cruz, sc-50164; OBSL1: Santa Cruz, sc-241578; SPAG4: Santa Cruz, sc-85927; SEPT12: Abnova, H00124404-B01P; LAMINB1: abcam, ab16048) for 60 min at room temperature. After being washed with TBS, the sections were exposed to the Alexa Fluor 568 donkey antigoat IgG antibody (Invitrogen, cat no. A-11057, USA), Alexa Fluor 488 donkey antimouse IgG antibody (Invitrogen, cat no. A-21202, USA), or Fluor 568 donkey antirabbit IgG antibody (Invitrogen, cat no. A-10042, USA) for 60 min at room temperature and washed again with TBS. A MitoTracker dye (Invitrogen, M7514, USA) and 4',6-diamidino–2-phenylindole (Invitrogen, D3571, USA) were used to stain the mitochondria and nuclei, respectively. The midpieces of the spermatozoa were stained using the mitochondrial tracker (Invitrogen, M7514, USA), and 4',6-diamidino–2-phenylindole (DAPI; Invitrogen, D3571, USA) was used to stain the nuclei. Labeled spermatozoa were examined and images were captured using the upright microscopy system BX60 (Olympus, Tokyo, Japan), and MetaMorph image analysis software was used to analyze the acquired images.

### Cloning and transfection

The full lengths of SEPT12 and SPAG4 were RT-PCR-amplified from a human RNA panel and cloned into the pEGFP-N1 and pFLAG-CMV2 vectors, as described previously [34]. All constructs were confirmed by performing DNA sequencing. After the cell line was transfected with plasmids by using Lipofectamine 2000 (Invitrogen, USA), the cells were subjected to a coimmunoprecipitation (Co-IP) assay and immunoblotting (IB).

### Coimmunoprecipitation assay

NTERA-2 d.D1 (NT2D1), a pluripotent human testicular embryonal carcinoma cell line, was used for the Co-IP experiment. The Co-IP analysis was performed according to the procedure described in our previous study [34]. The cell lysates obtained from cells transfected with the plasmids were precleared by incubating them with 50 μL of protein A⁄G beads (Santa Cruz, USA) for 1 h at 4°C on a rotator. The clear supernatant was incubated overnight with control IgG, anti-GFP (Santa Cruz, sc-9996, USA), or an anti-FLAG antibody (Sigma, F1804, USA; Sigma, F2555,USA). The samples were then washed twice with 1 × PBS and subjected to IB. The anti-FLAG (Sigma-Aldrich, F-1804, USA) and anti-LAMINB1 antibodies (Abcam, ab16048, UK) were used. Western blot analysis was performed according to the standard protocol [18].

## Results

### Screening proteins that interact with SEPT12

To dissect the possible molecular functions of SEPT12, we used a yeast 2-hybrid system to identify SEPT12 interactors. Over 100 clone sequences were sequenced. We identified 7 candidate proteins selected from the yeast 2-hybrid system according to the clone sequenced with high frequencies and expressed in human testis according to the UniGene profiles of NCBI, SEPT4, SEPT6, PRM2, SPAG4, NDC1, PCM1, and OBSL1 (Table 1). According to the functions and characteristics of the interactors, 3 subgroups were distinguished: SEPT family proteins (SEPT4 and SEPT6), nuclear or nuclear membrane proteins (PRM2, SPAG4, and NDC1), and sperm-related structural proteins (PCM1 and OBSL1).

**Table 1:** List of SEPT12-interacted proteins from yeast 2-hybrids.

Symbol	Gene	Function
**SEPT family proteins**		
SEPT6	*SEPTIN6*	Cell Cycle
SEPT4	*SEPTIN4*	Apoptosis, Sperm Annulus
**Nuclear/nuclear membrane proteins**		
PRM2	*Protamine 2*	Protamines are the major DNA-binding proteins in sperm nucleus.
SPAG4	*Sperm associated antigen 4*	Nucleus envelope protein; Sperm outer dense fiber-associated protein.
NDC1	*NDC1 transmembrane nucleoporin*	Transmembrane nucleoporin is required for nuclear pore complexes assemble.
**Sperm-related structural proteins**		
PCM1	*Pericentriolar material 1*	It is involved in centrosomal formation.
OBSL1	*Obscurin-like 1*	As a cytoskeleton protein and interacts with titin and ankyrin

### Expression patterns of SEPT12 interactors

**Fig 1:**
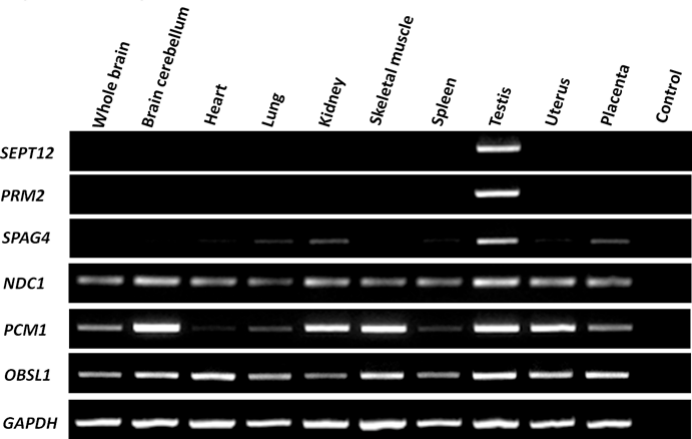
RT-PCR expression profiles of SEPT12 interactors in humans. The transcriptional expression of SEPT12, PRM2, SPAG4, NDC1, PCM1, and OBSL in human organs. GAPPDH was used as a loading control.

**Fig 2:**
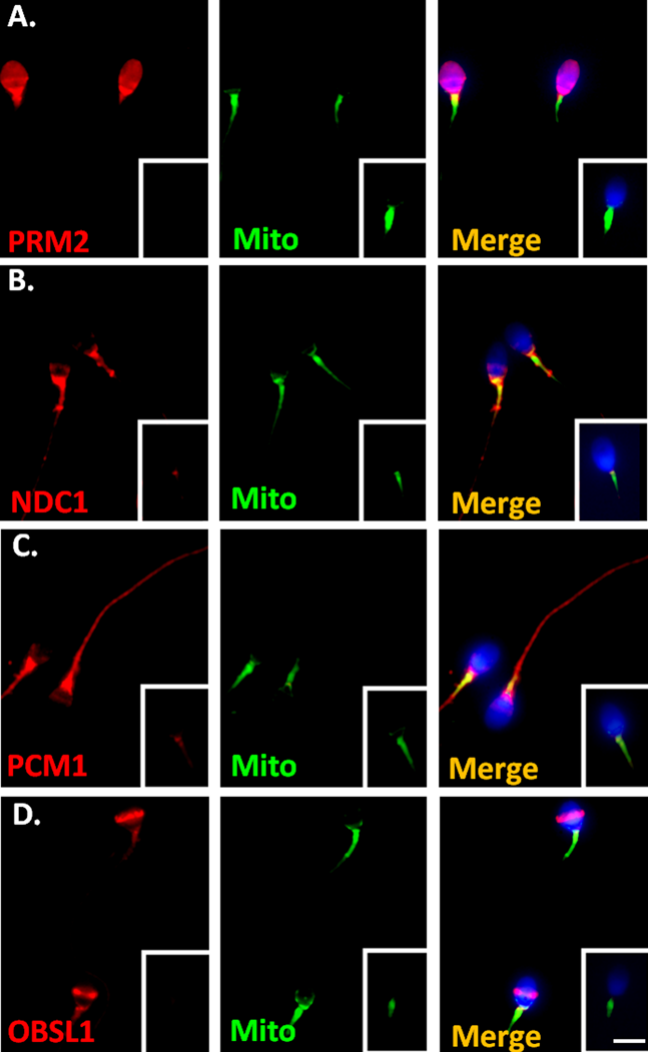
Localization of SEPT12 interactors in human-ejaculated spermatozoa. Immunofluorescence detection of (A) PRM2, (B) NDC1, (C) PCM1, and (D) OBSL in human-ejaculated spermatozoa. Left panel: anti-SEPT12-interactor antibody (red). Middle panel: MitoTracker (Mito; green). Right panel: combination of the left and middle panels. Figure A–D. Staining with control IgG is shown in the right lower corner. **More than 200 spermatozoa were evaluated at each antibody.** Scale bar: 5 μm.

**Fig 3:**
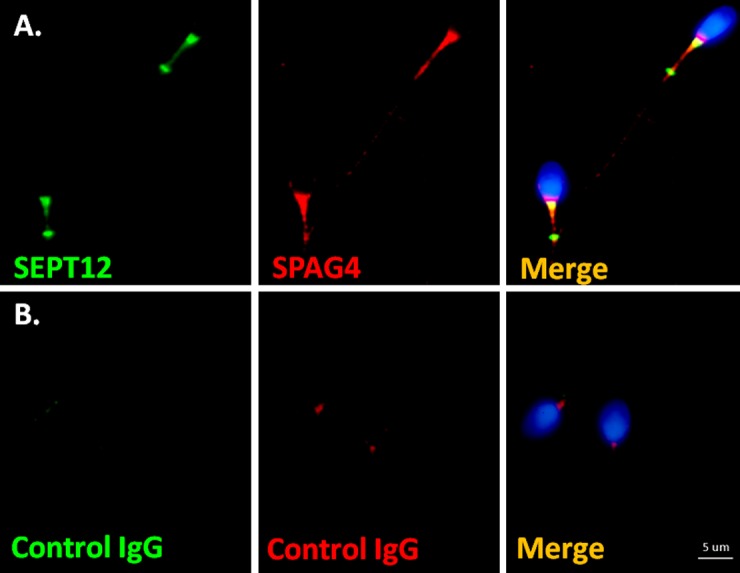
SEPT12 is colocalized with SPAG4 in human-ejaculated spermatozoa. (A) Immunofluorescence detection of SEPT12 (left panel; green) and SPAG4 (middle panel; red). Right panel: a combination of the left and middle panels. (B) Anticontrol IgG. **More than 200 spermatozoa were evaluated.** Scale bar: 5 μm.

The first interactor group comprised SEPT family proteins. According to relevant studies, including our own, SEPT1/4/6/7 complexes are involved in the structural integration of the sperm tail and serve as asthenozospermia markers [11–14,35]. We focused on the other interactors (PRM2, SPAG4, NDC1, PCM1, and OBSL1) in further study. To determine the expression patterns of these candidates, a human RNA panel was used to conduct RT-PCR. The interactors were highly expressed in the human testis (Fig. 1). In addition, PRM2 was localized in the head of human-ejaculated spermatozoa. NDC1 was primarily expressed in the sperm neck between the sperm head and tail, and in the midpiece regions of human-ejaculated spermatozoa. PCM1 was present in the sperm neck and in the entire sperm tail. OBSL1 was primarily located in the equatorial segment of the sperm head and was less prevalent in the sperm neck region (Fig. 2). Furthermore, SPAG4 was expressed in the sperm neck and midpiece regions and colocalized with SEPT12 expression (Fig. 3). These results indicated that all of the SEPT12 interactors were expressed in the testis and human-ejaculated spermatozoa; these results were similar to the expressional patterns of SEPT12.

### SPAG4 as a SEPT12 interactor

**Fig 4:**
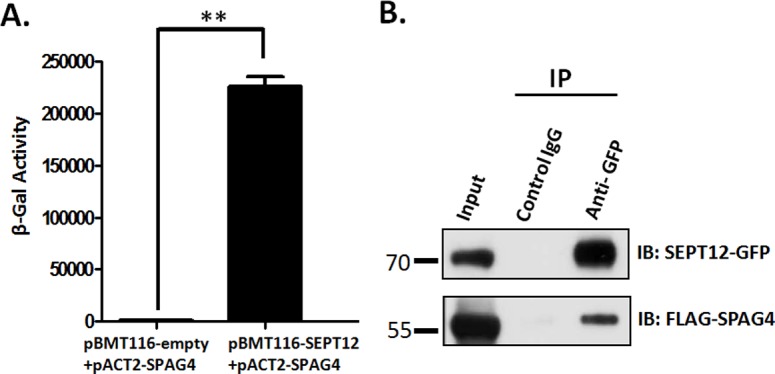
SEPT12 interacts with SPAG4. (A) β-galactosidase activity was quantified using yeasts containing pairs of baits and prey plasmids as indicated. Two-tailed Student’s *t* test; error bars indicate ±SEM. (B) Co-IP of FLAG-SPAG4 with SEPT12-GFP. The cell lysates of the NT2D1 cells, transfected with pEGFP-SEPT12 and a pFLAG-SPAG4 vector, are subjected to IP with an anti-GFP antibody (right panel, Row 3) or a nonspecific control IgG (right panel, Row 2) and immunoblotted (IB) with an anti-GFP or anti-FLAG antibody. An input protein (5%) was used as the control in the IB of the transfected cell lysates (right panel, Row 1).

In our previous studies, mutations of SEPT12 resulted in teratozospermia (e.g., abnormal sperm heads and sperms without tails) and DNA damage in human and mice spermatozoa [18,19]. In addition, the knockout of SPAG4 in *Drosophila* disturbed the sperm head morphology and caused dissociation of centrioles from the nucleus, resulting in the loss of connection between the sperm head and the tail [28]. According to similar phenotypes of SEPT12 in null mice and the knockout of SPAG4 in *Drosophila*, we suggest that the loss of SEPT12 affects the biological functionality of SPAG4 and results in teratozospermia in infertile men. First, to test whether SEPT12 interacts with SPAG4, we conducted a β-galactosidase assay of a yeast 2-hybrid model and Co-IP assay of NT2D1 cells. After transformation with SEPT12 and SPAG4 vectors,β-galactosidase activity was strongly induced through interaction between SEPT12 and SPAG4 (Fig. 4A). The NT2D1 cells were cotransfected with pEGFP-SEPT12-GFP and pFLAG-SPAG4 vectors and then subjected to immunoprecipitation (IP) with an anti-GFP antibody and control IgG. After IP, the samples were immunoblotted with an anti-GFP and anti-FLAG antibody, respectively. Fig. 4B shows that FLAG-SPAG4 was pulled down with SEPT12-GFP (Fig. 4B, **right row**; S1 Fig.). In addition, the cells transfected with pFLAG-SPAG4 and combined with a pEGFP-empty vector were subjected to a Co-IP protocol as a control (S2 Fig.). These models indicated that SEPT12 interacts with SPAG4.

### Dynamic expression of SEPT12 and SPAG4 during human spermiogenesis

**Fig 5:**
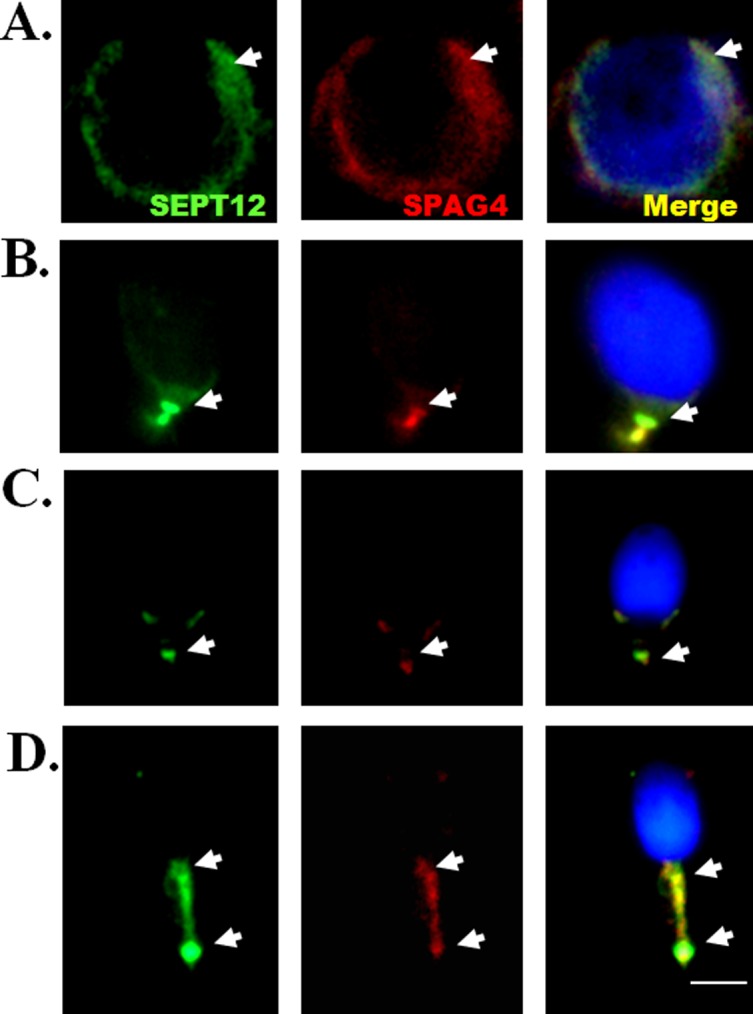
Localization of SEPT12 and SPAG4 during human spermiogenesis. IFA showed multiple localizations of SEPT12 and SPAG4 signals. A total of 32 male germ cells were evaluated as(A) Round spermatids (n = 7; labeled with anti-SEPT12 and anti-SPAG4 antibodies as the presented figure, 7/7); (B) elongating and (C) elongated spermatids (n = 12;12/12); (D) ejaculated spermatozoa (n = 13;12/13). (A–D) Anti-SEPT12 antibody (left panel; green), anti-SPAG4 antibody (middle panel; red), and a combination of the left and middle panels (right panel). The arrow indicates SEPT12 or SAPG4 signals. Scale bar: 5 μm.

To delineate the localization of SEPT12 and SPAG4 during human spermiogenesis, an IFA of human and murine testicular sections was performed. First, SPAG4 is specifically expressed in spermatids during murine spermiogensis (S3 Fig.). Second, we determined that SEPT12 was located at the nuclear periphery in the human round spermatid stage with SPAG4 (Fig. 5A and S4 Fig.). Furthermore, at the elongated spermatid stage, SEPT12 was colocalized with SPAG4 in the sperm neck regions (Fig. 5B and 5C). Finally, SEPT12 and SPAG4 colocalized in the midpiece region of human-ejaculated spermatozoa (Fig. 5D). These results indicated that SEPT12 colocalizes with SPAG4 during human spermiogenesis.

### SEPT12 interacts with SPAG4 /LAMINB1 complexes

**Fig 6:**
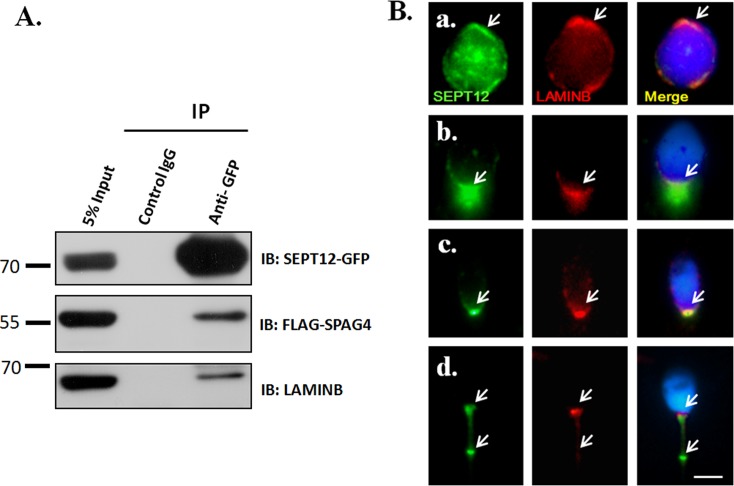
SEPT12 interacts and colocalizes with LAMINB1 male germ cells. **(A)** Co-IP of FLAG-SPAG4 and LAIMINB1 with SEPT12-GFP. The cell lysates of the NT2D1 cells, transfected with pEGFP-SEPT12 and a pFLAG-SPAG4 vector, are subjected to IP with an anti-GFP antibody (right panel, Row 3) or a nonspecific control IgG (right panel, Row 2), followed by IB with an anti-GFP, anti-FLAG, or anti-LAMINB1 antibody. An input protein (5%) was used as a control during the IB of the transfected cell lysates (right panel, Row 1). (B) An IFA revealed multiple localizations of SEPT12 and LAMINB1 signals. A total of 32 male germ cells were were evaluated as (A) Round spermatids (n = 9; labeled with anti-SEPT12 and anti-LAMINB1antibodies as the presented figure,9/9); (B) elongating and (C) elongated spermatids (n = 11;11/11); (D) ejaculated spermatozoa (n = 10;10/10). (A–D) Anti-SEPT12 antibody (left panel; green), anti-LAMINB1 antibody (middle panel; red), and a combination of the left and middle panels (right panel). The arrow indicates SEPT12 or LAMINB1 signals. Scale bar: 5 μm.

SPAG4 containing the SUN domain belongs to the SUN family as a nuclear membrane protein [36,37]. Regarding this family, the most detailed studies have examined SUN1 and SUN2, which interact with LAMIN through N-terminal domains and connect to cytoplasmic actin by direct interaction with Nesprin [22–24]. Furthermore, Fig. 4 shows that SEPT12 directly interacts with SPAG4. On the basis of previous evidence, we suggest that SEPT12/SPAG4 complexes also interact with LAMIN. The NT2D1 cells were cotransfected with pEGFP-SEPT12-GFP and pFLAG-SPAG4 vectors and then subjected to IP with an anti-GFP antibody and control IgG. After IP, these samples were immunoblotted with an anti-GFP antibody, anti-FLAG antibody, and anti-LAMINB1 antibody. The Co-IP experiments revealed that FLAG-SPAG4 and LAMINB1 were coimmunoprecipitated with SEPT12-GFP (Fig. 6A, **left**; S5 Fig.). In addition, SEPT12 was coexpressed with LAMINB1 in the nuclear periphery of round spermatids and slightly expressed in the sperm neck of elongating and elongated spermatids (Fig. 6B a–c). Finally, SEPT12 and LAMINB1 were slightly costained at the sperm neck, and SEPT12 was expressed only at the annulus of human-ejaculated spermatozoa (Fig. 6B d).

### SEPT12 mutations affect binding ability with SPAG4/LAMINB1 complexes

**Fig 7:**
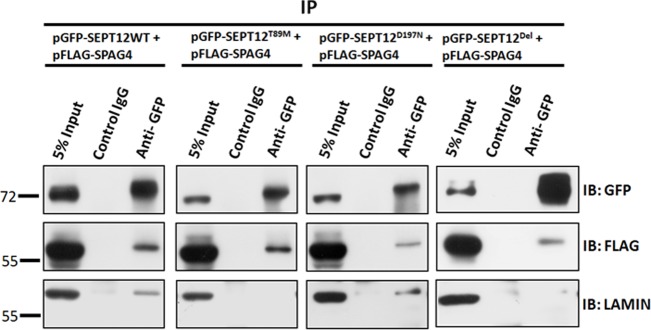
Mutated SEPT12 affects the interactions between SEPT12/SPAG4 /LAMIN complexes. SEPT12-WT (wild-type), SEPT12^T89M^ (Thr89Met), SEPT12^D197N^ (Asp197Asn), and SEPT12^Del^ (c.474G/A-induced deleted form) are shown in Panels 1, 2, 3, and 4, respectively. Co-IP of FLAG-SPAG4 and LAIMINB1 with SEPT12-GFP. The cell lysates of the NT2D1 cells, transfected with pEGFP-SEPT12 or mutated SEPT12 and a pFLAG-SPAG4 vector, are subjected to IP with an anti-GFP antibody (Row 3) or a nonspecific control IgG (Row 2), followed by IB with an anti-GFP, anti-FLAG, or anti-LAMINB1 antibody. An input protein (5%) was used as a control during the IB of the transfected cell lysates (Row 1).

Three mutated SEPT12 proteins that caused teratozospermia and oliogozospermia in our previous studies, SEPT12^T89M^ (Thr89Met), SEPT12^D197N^(Asp197Asn), and SEPT12^Del^ (c.474G/A-induced deleted form), were examined further in the current study [18,19]. After cells were cotransfected with pFLAG-SPAG4 and wild pEGFP-SEPT12 or mutated pEGFP-SEPT12 vectors, the cell lysates were subjected to a Co-IP assay. Fig. 7 shows that SEPT12^D197N^ and SEPT12^Del^ exhibited decreased ability to interact with SPAG4 compared with wild GFP-SEPT12. Furthermore, the SEPT^T89M^ and SEPT12^Del^ did not interact with LAMINB1. These results indicated that SEPT12 mutations affect the ability of SEPT12 to bind with SPAG4/LAMINB1 complexes. We suggest that loss of interactions between the SEPT12 and SPAG4/LAMINB1 complexes affects the integration of the nuclear envelope and eventually induces the dysregulation of sperm head formation and sperm DNA damage.

## Discussion

In this study, several SEPT12 interactors were identified and their expressions in human spermiogenesis were investigated. Three proteins, PRM2, NDC1, and SPAG4, are related to a nuclear or a nuclear membrane structure. SEPT12 interacts with the SAPG4/LAMINB1 complexes and colocalizes at the rim of the nuclear periphery during spermiogenesis. SEPT12 mutation resulted in loss of interaction between SEPT12 and SAPG4/LAMINB1 complexes. On the basis of these findings, we propose that the SEPT12/SPAG4/LAMINB1 complexes play a pivotal role in the formation of the nuclear envelop during human spermiogenesis.

### SEPT12 interactors identified from a yeast 2-hybrid system

This was the first study to identify SEPT12 interactors. 1)The 3 identified groups are the SEPT family proteins (SEPT4 and SEPT6), nuclear or nuclear membrane proteins (protamine2, NDC1 transmembrane nucleoprotein, and sperm-associated antigen 4), and sperm-related structural proteins (pericentriolar material 1 and obscurin-like 1) (Table 1). Considering that SEPT proteins form homomeric or heteromeric filament-like structures, such as the SEPT2/6/7, SEPT7/9b/11, and SEPT4/5/8 complexes, in several types of cells, we identified SEPT4 and SEPT6 as components of SEPT12 complexes [7,38,39]. SEPT4 and SEPT6 play functional roles in the annulus of mature spermatozoa [11,15,35]. 2) Nuclear or nuclear membrane proteins: PRM2 and PRM1 are major sperm proteins involved in the packing of nuclear DNA during spermiogenesis [40]. SPAG4 (SUN4) belongs to the SUN family [28]. One of the well-known proteins is SUN1, which is a nuclear membrane protein that links with LAMIN and the cytoplasmic cytoskeleton [23]. NDC1 is a transmembrane nucleoprotein that is involved in the assembling of nuclear pore complexes [41]. 3) Sperm-related structural proteins: PCM1 is a component of centriolar satellites, which are electron-dense granules scattered around the centrosomes [42]. Knockdown studies have demonstrated that this protein is essential for the correct localization of several centrosomal proteins and for anchoring microtubules to the centrosomes [42]. OBSL1 is a cytoskeletal adaptor protein that links the internal cytoskeleton of cells to the cell membrane [43]. In this study, we also characterized the expressional patterns of NDC1, PCM1, SPAG4, PCM1, and OBSL1 in the human-ejaculated spermatozoa. NDC1, SPAG4, PCM1, and OBSL1 were expressed in the sperm neck, tail, or equatorial segment of the sperm (Fig. 2). On the basis of the characteristics of these SEPT12 interactors, we suggest that they coregulate sperm head formation and tail elongation during human spermiogenesis.

### SEPT12/SPAG4/LAMINB1 complexes during human spermiogenesis

In *Drosophila*, knockout of SPAG4 disturbed the sperm head morphology and caused dissociation between the nucleus and the centrosome, a polymerizing initiation site of the sperm tail [28]. This result suggests that SPAG4 is critical for the morphological shaping of the sperm head, the formation of the nuclear membrane, and the connection between the sperm head and tail in spermiogenesis. Shao et al. indicated that SPAG4 localizes to the microtubules of the manchette and axoneme during rat spermiogenesis and interacts with Odf1, a prominent structural protein of the midpiece and principal piece in spermatozoa [30]. In a transiently transfected cell model, SPAG4-GFP localized at the NE and ER [31]. In this study, we determined that SEPT12, SPAG4, and LAMINB1 formed complexes and exhibited similar dynamic expression patterns during human spermiogenesis. SEPT12 cooperated with LAMINB1 through SPAG4 and at the rim of the nuclear periphery in round spermatids. Furthermore, at the elongation spermatids, elongated spermatids and human-ejaculated spermatozoa, SEPT12/SPAG4 maintained the link between the sperm neck, centrosome, and the axoneme, is a main structural structure of sperm tail. These results suggesting that SPAG4 is a linker proteins between LAMIN and the cytoplasmic cytoskeleton and this conclusion is consistent with the results of previous studies [22–24].

### Mutated SEPT12 affects binding between SEPT12/SPAG4/LAMINB1 complexes

The sperm of mice and humans with defective *SEPT12/Sept12* exhibited distinctive morphological defects (e.g., abnormal head and bent tail, premature chromosomal condensation, and a damaged nucleus) [15,17–19]. In addition, oocytes injected with sperm carrying abnormal SEPT12 cannot ould not develop into the morula stage because of severe DNA damage [17]. Recently, several studies have indicated that SEPTs are related to cell cycle arrest and DNA-damaged pathways. First, during cell cycle progression, SEPT2 interacts with centromere-associated protein E, a microtubule motor protein, to stabilize the binding between kinetochore and spindle microtubules [44,45]. Loss of SEPT2 results in chromosome loss from the metaphase plate, a lack of chromosome segregation and spindle elongation, and incomplete cytokinesis. Second, as Kremer et al. indicated, SEPTs are related to DNA damage through the response of the SOCS7 pathway to UV damage from NCK accumulation [46,47]. In this study, we determined that SEPT12 interacts with SPAG4/LAMINB1 complexes and is involved in the formation of the nuclear membrane. In addition, SEPT12 mutation reduced the ability of SEPT12 to bind with SPAG4/LAMINB1 complexes (Fig. 7). Considering this, we suggest that the disruption of complexes by mutated SEPT12 results in morphological defects in the nuclear envelope of sperm, inducing DNA damage in sperm that may be mediated by the SOCS7 pathway. We suggest that the spermatozoa with this mutated SEPT12 cause the developmental arrest of the embryo, even when IVF and ICSI are used.

In this study, we determined several SEPT12 interactors active at various stages during human spermiogenesis. One of the interactors, SPAG4, is critical for the formation of the nuclear membrane of sperm and DNA integration in the differentiation of male germ cells.

## Supporting Information

S1 Fig Co-IP of FLAG-SPAG4 with SEPT12-GFP. The cell lysates of the NT2D1 cells, transfected with pEGFP-SEPT12 and a pFLAG-SPAG4 vector, are subjected to IP with an anti-GFP antibody (right and left panels, Row 3) or a nonspecific control IgG (right and left panels, Row 2), followed by IB with an anti-GFP (left panel) or anti-FLAG (right panel) antibody. An input protein (5%) was used as the control during the IB of the transfected cell lysates (right panel, Row 1). The marked region is shown in Fig. 4B.
(TIF)

S2 Fig Control for Co-IP: NT2D1 cell transfected with pFLAG-SPAG4 and a pEGFP-empty vector. The cell lysates of the NT2D1 cells, transfected with only a pFLAG-SPAG4 vector, are subjected to IP with an anti-GFP antibody (right and left panels, Row 3) or a nonspecific control IgG (right and left panels, Row 2), followed by IB with an anti-FLAG (left panel) or anti-GFP (right panel) antibody. An input protein (5%) was used as the control during the IB of the transfected cell lysates (right and left panels, Row 1).
(TIF)

S3 Fig IFA of SPAG4 in the seminiferous tubule of the mouse testis. SPAG4 (green) was expressed in spermatids and elongated spermatids labelled with Lectin (red; acrosome marker). General view of the mouse testis (A–D): (A) staining with SPAG4 (green) and (B) SPAG4 with acrosome marker (red, Lectin), respectively; (C) staining with control IgG and (D) IgG with acrosome marker (red, Lectin), respectively; (E) stage I–III; (F) stages IV–V, stages VII–VIII, and stages X–XII of murine spermatogenesis.
(TIF)

S4 Fig IFA of SPAG4 in the seminiferous tubule of the human testis. (A) SPAG4 (green) was expressed in spermatids and elongating spermatids. (B) Merge with (A) and DAPI; (C) staining with Control IgG (green). (D) Merge with (C) and DAPI. (Arrow head: spermatocytes; Arrows: spermatids).
(TIF)

S5 Fig Co-IP of FLAG-SPAG4 and LAIMINB1 with SEPT12-GFP. The cell lysates of the NT2D1 cells, transfected with pEGFP-SEPT12 and a pFLAG-SPAG4 vector, are subjected to IP with an anti-GFP antibody (right, middle, and left panels, Row 3) or a nonspecific control IgG (right, middle, and left panels, Row 2), followed by IB with an anti-GFP, anti-FLAG, or anti-LAMINB1 antibody. An input protein (5%) was used as a control during the IB of the transfected cell lysates (right panel, Row 1). The marked region is shown in Fig. 6A.
(TIF)
