# Supplementary figures and images for: SEPT12/SPAG4/LAMINB1 Complexes Are Required for Maintaining the Integrity of the Nuclear Envelope in Postmeiotic Male Germ Cells
Source: PLoS One. 2015 Mar 16;10(3):e0120722. doi: 10.1371/journal.pone.0120722 (PMC4361620; doi:10.1371/journal.pone.0120722)

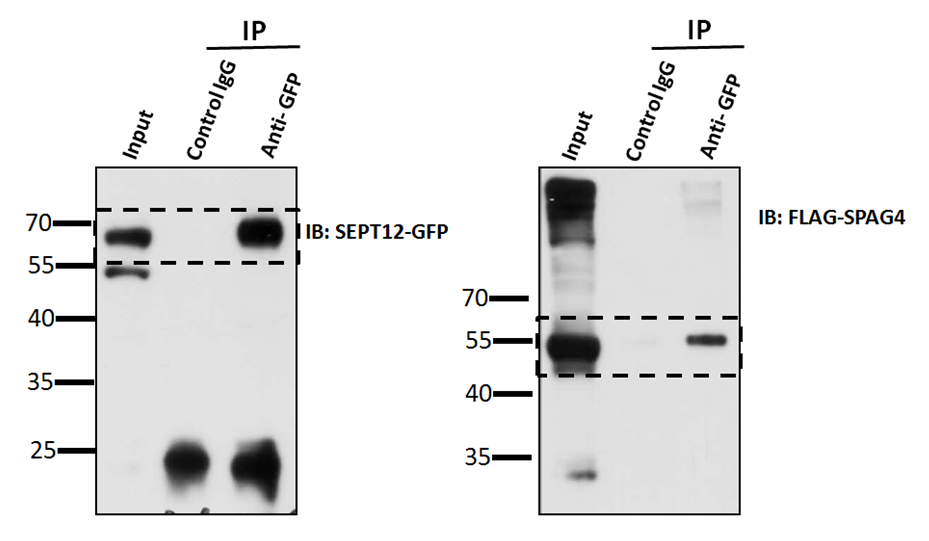

Supplement: S1 Fig — The cell lysates of the NT2D1 cells, transfected with pEGFP-SEPT12 and a pFLAG-SPAG4 vector, are subjected to IP with an anti-GFP antibody (right and left panels, Row 3) or a nonspecific control IgG (right and left panels, Row 2), followed by IB with an anti-GFP (left panel) or anti-FLAG (right panel) antibody. An input protein (5%) was used as the control during the IB of the transfected cell lysates (right panel, Row 1). The marked region is shown in Fig. 4B. (TIF) [file pone.0120722.s001.tif]

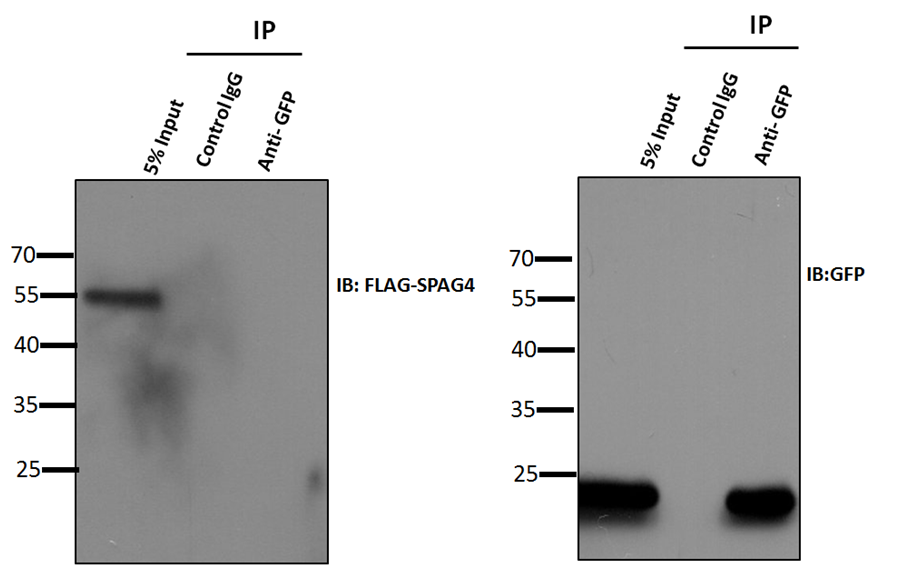

Supplement: S2 Fig — The cell lysates of the NT2D1 cells, transfected with only a pFLAG-SPAG4 vector, are subjected to IP with an anti-GFP antibody (right and left panels, Row 3) or a nonspecific control IgG (right and left panels, Row 2), followed by IB with an anti-FLAG (left panel) or anti-GFP (right panel) antibody. An input protein (5%) was used as the control during the IB of the transfected cell lysates (right and left panels, Row 1). (TIF) [file pone.0120722.s002.tif]

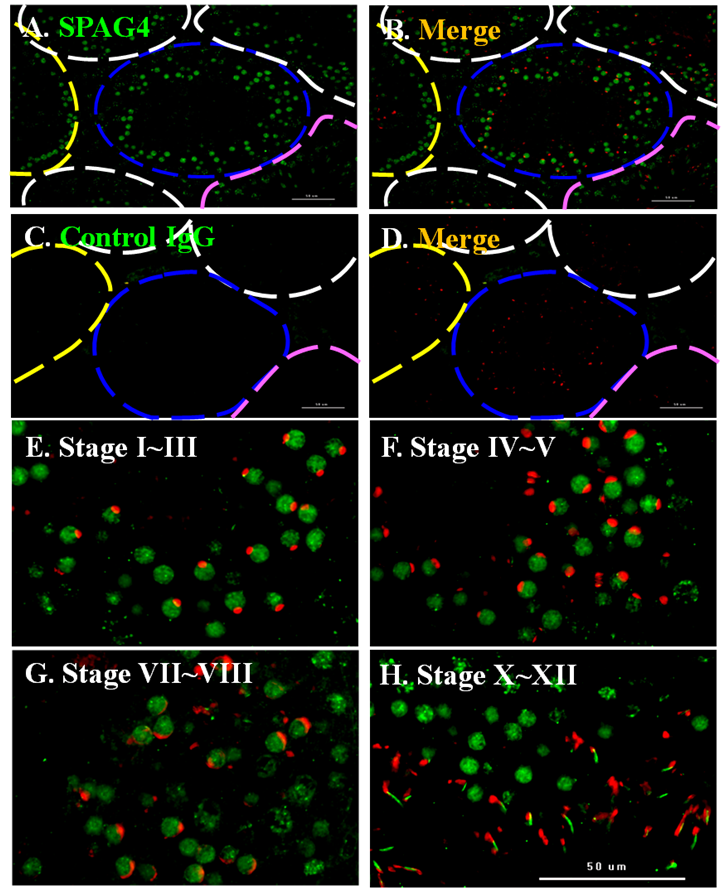

Supplement: S3 Fig — SPAG4 (green) was expressed in spermatids and elongated spermatids labelled with Lectin (red; acrosome marker). General view of the mouse testis (A–D): (A) staining with SPAG4 (green) and (B) SPAG4 with acrosome marker (red, Lectin), respectively; (C) staining with control IgG and (D) IgG with acrosome marker (red, Lectin), respectively; (E) stage I–III; (F) stages IV–V, stages VII–VIII, and stages X–XII of murine spermatogenesis. (TIF) [file pone.0120722.s003.tif]

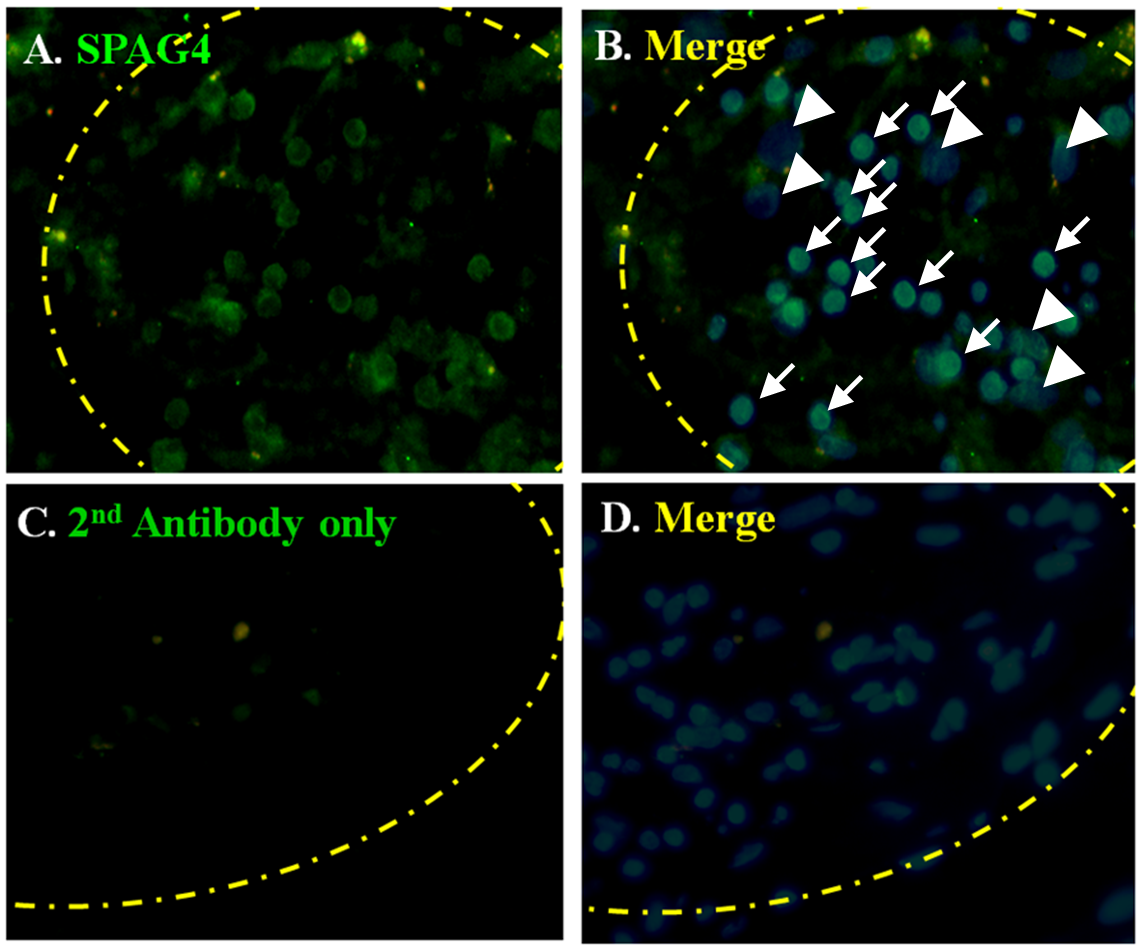

Supplement: S4 Fig — (A) SPAG4 (green) was expressed in spermatids and elongating spermatids. (B) Merge with (A) and DAPI; (C) staining with Control IgG (green). (D) Merge with (C) and DAPI. (Arrow head: spermatocytes; Arrows: spermatids). (TIF) [file pone.0120722.s004.tif]

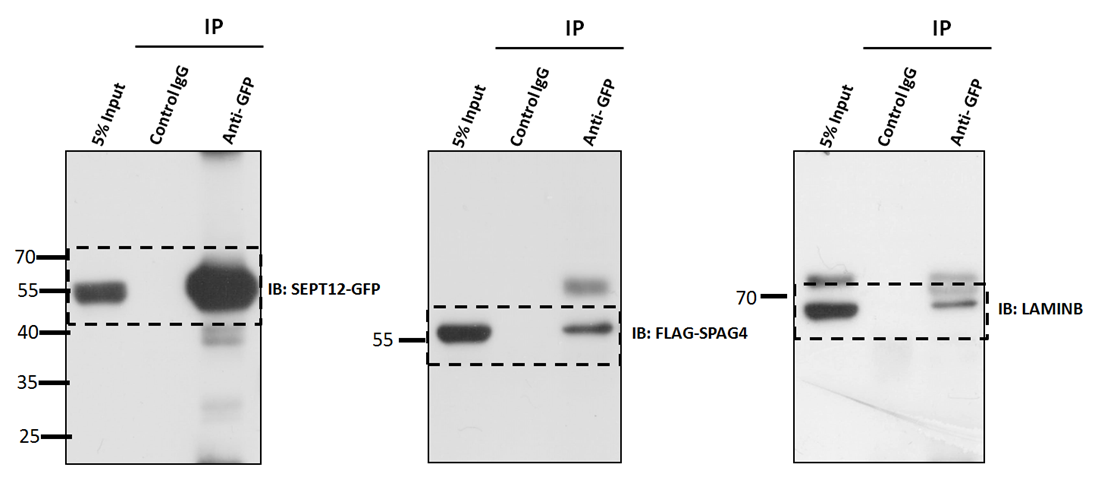

Supplement: S5 Fig — The cell lysates of the NT2D1 cells, transfected with pEGFP-SEPT12 and a pFLAG-SPAG4 vector, are subjected to IP with an anti-GFP antibody (right, middle, and left panels, Row 3) or a nonspecific control IgG (right, middle, and left panels, Row 2), followed by IB with an anti-GFP, anti-FLAG, or anti-LAMINB1 antibody. An input protein (5%) was used as a control during the IB of the transfected cell lysates (right panel, Row 1). The marked region is shown in Fig. 6A. (TIF) [file pone.0120722.s005.tif]
